# The Modifying Effects of Lifestyle Behaviors on the Association Between Drinking Water Micronutrients and BMI Status Among Children and Adolescents Aged 7~17: A Population-Based Regional Surveillance in 2022

**DOI:** 10.3390/nu16223931

**Published:** 2024-11-18

**Authors:** Manman Chen, Xiuhong Zhang, Jianuo Jiang, Tian Yang, Li Chen, Jieyu Liu, Xinli Song, Yi Zhang, Ruolin Wang, Yang Qin, Ziqi Dong, Wen Yuan, Tongjun Guo, Zhiying Song, Jun Ma, Yanhui Dong, Yi Song, Yuhan Qin

**Affiliations:** 1 School of Population Medicine and Public Health, Chinese Academy of Medical Sciences and Peking Union Medical College, Beijing 100730, China; chenmm@pumc.edu.cn; 2 Public Health Institute of Inner Mongolia Center for Disease Control and Prevention, Hohhot 010031, China; zhangxh519@126.com (X.Z.); nmyangt@163.com (T.Y.); 3 National Health Commission Key Laboratory of Reproductive Health, Institute of Child and Adolescent Health, School of Public Health, Peking University, Beijing 100191, China; 1810306143@pku.edu.cn (J.J.); clcl@bjmu.edu.cn (L.C.); jieyulynne@163.com (J.L.); lilidaren1996@bjmu.edu.cn (X.S.); zhangyee@bjmu.edu.cn (Y.Z.); 2411110217@bjmu.edu.cn (R.W.); thomasqin@bjmu.edu.cn (Y.Q.); 1910306215@pku.edu.cn (Z.D.); yuanwen@bjmu.edu.cn (W.Y.); guotj@bjmu.edu.cn (T.G.); 2316394116@bjmu.edu.cn (Z.S.); majunt@bjmu.edu.cn (J.M.); songyi@bjmu.edu.cn (Y.S.)

**Keywords:** drinking water micronutrients, BMI, children and adolescents, lifestyle behaviors, large regional surveillance

## Abstract

Background: This study aims to investigate the potential modifying effects of lifestyle behavior on the association between drinking water micronutrients and body mass index (BMI) in a large population of children and adolescents. Methods: Data of the present analysis came from a comprehensive regional large-scale surveillance study in 2022, involving 172,880 children and adolescents (50.71% boys vs. 49.29% girls) aged seven to seventeen. A restricted cubic spline (RCS) analysis was utilized to examine the exposure-response association of regular drinking water indices (including fluoride, nitrate nitrogen, pH, chloride, sulfates, and total dissolved solids (TDS), total hardness (TH), and chemical oxygen demand (COD)) with BMI. Generalized linear model and logistic regression were conducted to relate BMI and quartiles of drinking water micronutrients. Results: Our findings reveal a nonlinear association between nitrate nitrogen (P for nonlinear < 0.001) and pH (P for nonlinear < 0.001) with BMI. High TH and COD levels significantly increase BMI. Notably, fluoride and chloride were associated with BMI Z-scores but not with overweight and obesity (OB). The BMI Z-score showed a more pronounced association with low and high pH levels in girls. For urban participants, increased TH levels were associated with a higher risk of OB. This study also found that adopting healthy lifestyles could mitigate the negative effects of fluoride, chloride, and sulfate on BMI Z-scores. Conclusions: This large surveillance study provides new insights into the complex interplay between drinking water micronutrients and BMI in children and adolescents. The association of various drinking water parameters on BMI varies, necessitating ongoing focus on their effects, particularly among girls and urban individuals. Healthy lifestyle behavior could mitigate the effects of fluoride, chloride, and sulfate on BMI Z-score.

## 1. Introduction

Water is a crucial nutrient that plays a key role in the body’s chemical and biochemical reactions and is essential for maintaining human health [1,2]. With the rapid development of population growth, industrial and agricultural production activities, and urbanization in modern society, limited water resources and the water environment have had a huge impact. Studies have shown that long-term drinking of water of poor quality may cause cardiovascular and cerebrovascular sclerosis, digestive system diseases, mental health, and cancer [3,4,5]. Approximately one-quarter of the global population lacks access to safe drinking water, leading to diseases that result in about 1.4 million deaths annually due to inadequate water quality, sanitation, and hygiene. The World Health Organization (WHO) attributes 80% of global diseases to impure drinking water, and half of the worldwide child mortality rate is associated with the consumption of contaminated water [6]. In 2019, the United Nations Children’s Fund and the WHO reported that an estimated 2.2 billion people globally do not have access to safely managed drinking water services, with around 785 million individuals lacking even basic drinking water services [7]. In addition, regular monitoring of drinking water quality is essential to detect potential contamination in potable water supplies [8]. This highlights the urgent need for regular monitoring of water quality to prevent the long-term effects of drinking water contamination on human health.

The indicators of drinking water are of utmost importance for human health, influencing well-being at all life stages [9,10]. The presence of chemical and biological contaminants in water, particularly when their concentrations exceed the limits set by safety standards, can lead to increased health risks [11]. For instance, diarrhea, often stemming from infections due to oral-fecal contamination, is the second most prevalent cause of mortality among children under five years old, especially in regions such as Africa and South Asia [12]. Current drinking water standards fail to address the long-term health implications for vulnerable populations, particularly children and adolescents. Rather than discussing health issues beyond standard water sources, focus on the potential health risks posed by water indicators within the standards of existing drinking water for these vulnerable populations.

The issue of rising overweight and obesity rates among Chinese children and adolescents has become a critical public health concern. A national survey in China reported that the prevalence of overweight students showed a significant rise from 4.3% in 1995 to 18.4% in 2014 [13]. Given that BMI is also closely related to environmental factors, notably the impact of drinking water contamination [14]. Several studies have highlighted the potential health risks associated with exposure to manganese in drinking water, particularly concerning neurodevelopmental outcomes in children [15]. Meanwhile, exposure to arsenic in drinking water has also been linked to a range of adverse health effects, including cardiometabolic and neurodevelopmental impairments in children [16,17,18]. A recent study found that perchlorate exposure in drinking water had a negative dose-response relationship with the BMI of children and adolescents [6]. Additionally, a cross-sectional study [19] demonstrated that children from households without treated drinking water were at a considerably increased risk of stunting. BMI has been associated with increased all-cause and cardiovascular mortality. According to statistics from the Global Burden of Disease (GBD) Study 2019, high BMI was the top five leading risk factors for cardiovascular diseases [20]. However, there has been insufficient evidence regarding the associations between regular monitoring indicators of drinking water and BMI in children and adolescents.

A significant increasing trend has been witnessed over the past three decades; considering the high obesity burden nowadays with the largest number of obese children in China, the obesogenic environmental factors should be paid attention to. Based on extensive regional, large geographic, and population-scale administrative surveillance of over 170,000 children and adolescents and over 5000 drinking water samples, we investigated the association between drinking water indicators and BMI in children and adolescents aged 7~17 years. Considering the insufficient research in developing countries, this study aimed to provide crucial empirical evidence on the associations of multiple drinking water indicators with BMI among children and adolescents and the effect of modification by lifestyle behavior to inform public health policies and interventions in China.

## 2. Materials and Methods

### 2.1. Study Design and Population

The present study, conducted in 2022, pertains to the monitoring participants in the “National Student Common Diseases and Health Influencing Factors Monitoring” program within the Inner Mongolia Autonomous Region in China. This common disease monitoring initiative is initiated every September and includes all 12 league cities, 103 banners, counties, and districts within the Inner Mongolia Autonomous Region. We employed a multi-stage cluster sampling method for school selection. Specifically, in each urban area, two primary schools, two junior high schools, two general high schools, one vocational high school, and one comprehensive university are randomly selected. In each suburban county, two primary schools, two junior high schools, and one high school are randomly selected. Within each selected school, two or more classes were chosen, and all students within these classes were included as a unit, ensuring a minimum of 80 students from each grade. Any remaining spots are filled by students from nearby equivalent schools. Overall, the sampling approach was designed to conduct a comprehensive and representative sampling of students for the monitoring process.

### 2.2. Geographical Location and Matching

**Figure 1:**
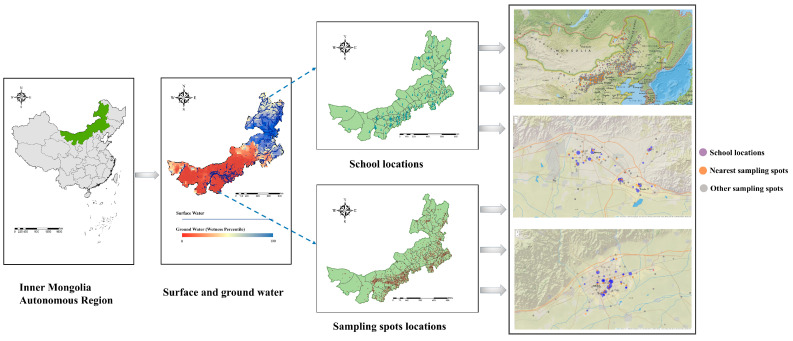
Spatial distribution of school and sampling spot locations in the study. Note: Groundwater data was acquired from https://nasagrace.unl.edu (accessed on 10 March 2024) in collaboration with the National Drought Mitigation Center, and surface water data was sourced from OpenStreetMap (https://www.openstreetmap.org, accessed on 10 March 2024).

The Inner Mongolia Autonomous Region, situated on China’s northern border, extends across the regions of Northeast, North, and Northwest China. Inner Mongolia boasts a notably elongated and narrow geographic expanse. First, using data from the National Student Common Diseases and Health Influencing Factors Monitoring, the reported school addresses of individual students and water sampling points are geocoded to extract the longitude and latitude coordinates. Second, a proximity-based method was used to match the school addresses to the nearest water sampling locations. The median distance between the school sites and water sampling points was 0.54 km (interquartile range: 0.26 km~1.09 km; range: 0 km~6.91 km) (Figure 1). Every eligible participant aged seven to seventeen years old in our study underwent a comprehensive physical examination as a prerequisite before data collection. Throughout this study, we excluded two distant monitoring stations from the analysis: Jinhe Middle School in Genhe City, Hulunbuir City, and Xing’an Mengke Right School. Additionally, Zhongqi Bayanhushu No. 4 Middle School was also excluded from the analysis.

Prior to the commencement of this study, all participants and their legal guardians provided their signed informed consent forms. This study received official approval from the Ethics Committee of the Inner Mongolia Autonomous Region Center for Disease Control and Prevention (approval code: 202309271; approval date: 27 September 2023).

### 2.3. Drinking Water Micronutrients

Drinking water monitoring in China is conducted by the Centers for Disease Control and Prevention. The water indicators monitoring data for the Inner Mongolia Autonomous Region in 2022 were retrieved from the National Drinking Water Quality and Hygiene Monitoring Information System, encompassing 104 districts and counties. A total of 5050 tap water samples were collected and examined following administrative standard methods (GB/T 5750-2006 [21], GB/T 5749-2006 [22], accessible online at https://openstd.samr.gov.cn/bzgk/gb/index, accessed on 10 March 2024). The methods were designed to apply the quality assessment of drinking water with clear fundamental principles, procedures, and criteria for the proper collection, preservation, quality control, and laboratory examinations.

In this study, we retained indicators that exhibited less than 10% missing data, encompassing eight parameters: fluoride, nitrate, pH, chloride, sulfate, total dissolved solids (TDS), total hardness (TH), and chemical oxygen demand (COD). The measurements for fluoride, nitrate, chloride, and sulfate were conducted through ion chromatography, with respective lower detection limits of 0.1 mg/L, 0.15 mg/L, 0.15 mg/L, and 0.75 mg/L. The pH levels were determined using the glass electrode method, achieving a precision of 0.01. TDS was quantified through the 105 °C drying and weighing method, with a detection threshold of 3.41 mg/L. TH was assessed using the disodium ethylene-diamine-tetra-acetate (EDTA) titration method, with a lower detection limit of 1.0 mg/L. The COD was ascertained through either the acidic or alkaline potassium permanganate titration method, with a detection limit of 0.05 mg/L. For indicators not detected, imputations were made by assuming the value of the lower detection limit divided by the square root of 2. More detailed information on the water sampling and examinations is provided in Supplementary Materials (Method S1). Information on water quality standard limits is provided in Table S1.

### 2.4. Outcome Measurements and Classifications

All participants underwent a comprehensive physical examination administered by medical personnel following a standardized protocol [23]. Height was measured using a calibrated mechanical stadiometer (model TZG, manufactured by Jiangyin No. 2 Medical Equipment Factory in Jiangsu, China), ensuring precision to within 0.1 cm. Similarly, weight measurements were carried out using a uniform and calibrated electronic scale (model RGT-140, produced by Shanghai Dachuan Electronic Weighing Apparatus Co. Ltd. in Shanghai, China), with precision to within 0.1 kg, and this was done while participants were dressed in lightweight attire. Body mass index (BMI) was computed by dividing an individual’s weight in kilograms by the square of their height in square meters (kg/m^2^). For each participant, an age- and sex-standardized BMI score (BMI Z-score) was calculated, referencing the standard population defined by the World Health Organization (WHO) [24]. The BMI Z-score calculations were performed using the ‘zanthro’ module in STATA 14.0, which can calculate z-scores for anthropometric measures in children and adolescents according to the WHO reference growth charts. The nutritional status was assessed based on the BMI Z-scores: A BMI Z-score of ≤1 indicated non-overweight and obesity (non-OB), while a BMI Z-score of >1 signified overweight and obesity (OB).

### 2.5. Questionnaires

The “Student Health Status and Influencing Factors Questionnaire” was used to gather demographic information (sex, birthdate, and residence) and lifestyle data, including vegetables (frequency of eating vegetables in the past seven days), fruits (frequency of eating fruits in the past seven days), sugared beverages (frequency of drinking sugared beverages in the past seven days), fried food (frequency of eating fried food in the past seven days), physical activity time (frequency of moderate to high-intensity physical activity for 60 minutes or more in the past seven days), daily outdoor time, daily sleeping time, smoking (yes/no), and alcohol drinking (yes/no). Information on students in grades one to three of primary school was collected from their legal guardians. According to the American Heart Association’s Strategic Impact Goals for 2020 [25], this study identified a total of nine key components for healthy lifestyles (vegetables > one time, fruits > one time, sugared beverages > one time, fried food > one time, physical activity times > one day, outdoor time > two hours, sleeping time > nine hours, non-smoking, and non-drinking). A healthy lifestyle is defined as meeting six or more of these nine criteria.

### 2.6. Statistical Analysis

Characteristics for participants with non-OB and OB groups were reported as percentages or mean (standard deviation, SD). The subgroup differences were examined using the student *t*-test (continuous variables) and Chi-squared test (categorical variables). We utilized box plots to compare the variations in regular drinking water micronutrients across diverse nutritional statuses. The subgroup differences in drinking water indicators were examined using the Mann–Whitney U-test.

To explore the potential non-linear associations of regular indices for drinking water with BMI Z-score and different nutritional status [6], we plotted the exposure-response curves with a restricted cubic spline (RCS) function with three knots (the percentiles were set to be 25th, median, and 75th) [26]. Additionally, this study controlled the age, sex, administrative region (urban/rural), vegetables, fruits, sugared beverages, fried food, physical activity time, smoking, drinking, outdoor time, and sleeping time variables in the models to control for potential confounding. Stratified analyses based on participants’ sex (girls/boys), living regions (urban/rural), and lifestyle behavior were also conducted to evaluate the disparities in water indicators (BMI) associations across participants and regions.

**Figure 2:**
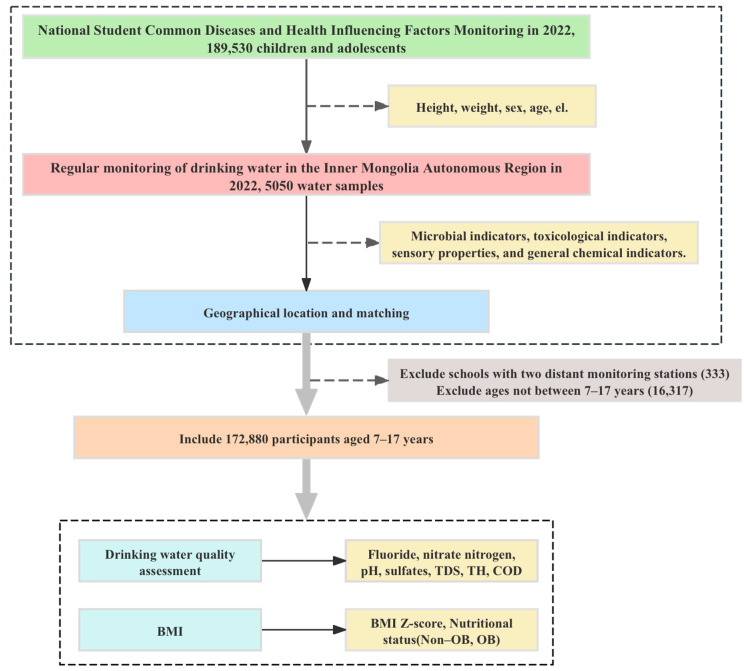
A flow chart illustrating the sequence of processes followed to analyze drinking-water samples.

The flow chart for data analyses is shown in Figure 2. All data analyses were performed using Stata 14.0 (College Station, TX, USA) and R software (version 4.3.0). Spatial analyses were conducted using ArcGIS 10.8. Two-sided *p* < 0.05 was considered as statistically significant.

## 3. Results

### 3.1. Characteristics of the Study Participants

Demographic characteristics of 172,880 participants (50.71% boys vs. 49.29% girls) are presented in Table 1. A total of 102,212 non-OB and 70,668 OB were included in this study, with an average BMI of 17.92 kg/m^2^ and 24.56 kg/m^2^ and an average BMI Z-score of −0.21 and 2.06, respectively. The age of the participants ranged between 12.33 (SD = 3.08) and 11.74 (SD = 2.81) years, with a significant difference between non-OB and OB groups. Significant differences in sex, age, height, weight, BMI, and BMI Z-score were observed between non-OB and OB groups (*p* < 0.001).

### 3.2. Description of Regular Indicators for Drinking Water

**Figure 3:**
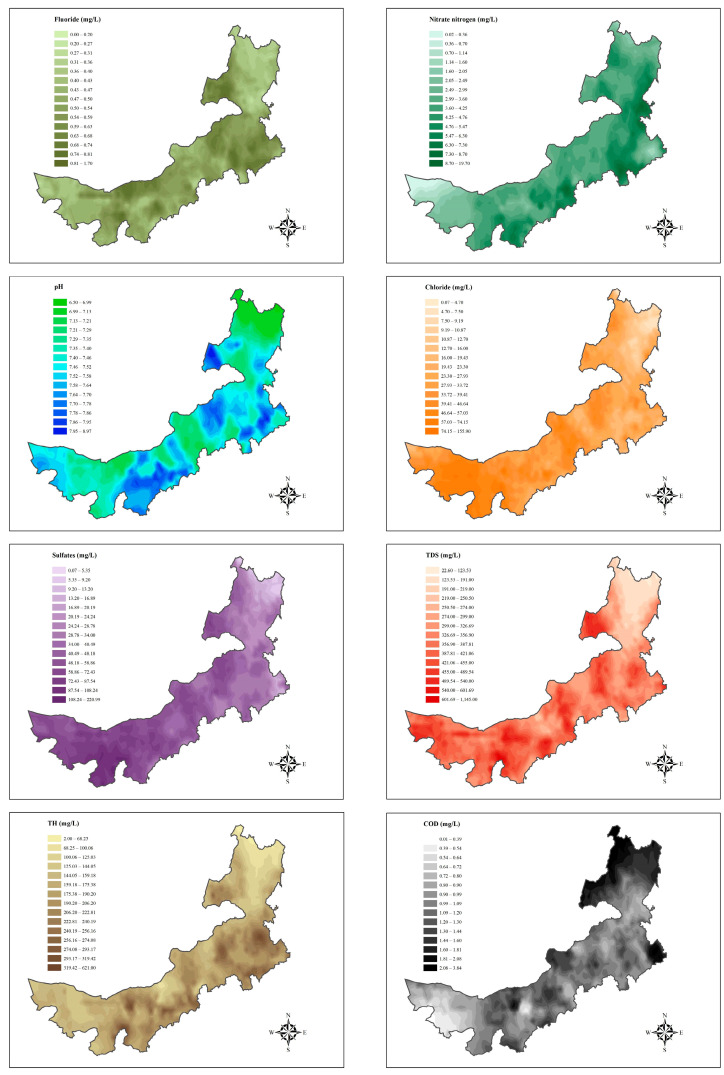
The spatial distribution of concentration of regular indices for drinking water in the Inner Mongolia Autonomous Region. Note: TDS, Total dissolved solids; TH, Total hardness; COD, Chemical oxygen demand.

The monitoring data of regular indicators in drinking water in the Inner Mongolia Autonomous Region are presented in Figure 3. Table S1 presents the main standard parameters for drinking water, while Figure S1 illustrates the characteristics of these parameters after statistical analysis. The median (IQR) for fluoride was 0.48 (0.30, 0.67) mg/L, nitrate nitrogen was 3.00 (0.80, 6.96) mg/L, pH was 7.50 (7.24, 7.81), chloride was 25.50 (10.40, 63.00) mg/L, sulfates was 35.42 (17.80, 93.27) mg/L, TDS was 326(252, 531) mg/L, TH was 214.50 (144.10, 286.97) mg/L, and COD was 0.96 (0.56, 1.44) mg/L, respectively. We identified significant differences in nitrate nitrogen, chloride, sulfates, TDS, TH, and COD between the non-OB and OB groups (*p* < 0.05). However, no significant differences were observed in fluoride and pH (*p* > 0.05). In addition, Table S2 presents the characteristics of drinking water indicators for drinking water in urban and rural areas. We found higher nitrate nitrogen and lower pH, chloride, sulfates, TDS, and TH levels in rural areas (*p* < 0.05).

### 3.3. Association Between Regular Drinking Water Micronutrients and BMI

**Figure 4:**
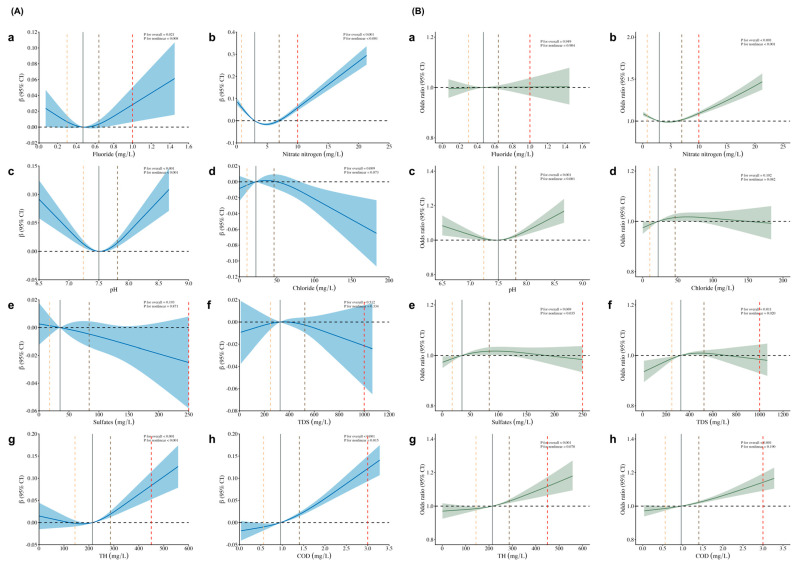
Exposure-response curves of the associations of regular drinking water indicators with BMI Z-score and OB of all participants. Note: (**A**), BMI Z-score; (**B**), OB; (**a**), fluoride; (**b**), nitrate nitrogen; (**c**), pH; (**d**), chloride; (**e**), sulfates; (**f**), TDS; (**g**), TH; (**h**), COD; OB, overweight and obesity; TDS, total dissolved solids; TH, total hardness; COD, chemical oxygen demand. Estimates were adjusted for sex, age, city-rural water, vegetables, fruits, sugared beverages, fried food, physical activity time, outdoor time, sleeping time, smoking, and drinking. Solid lines were predicted curves; shadow parts were 95% confidence intervals. The orange, black, and grey lines indicate the 25th, median, and 75th concentration levels. The red broken lines indicate the water quality standard limits according to the administrative standard.

**Figure 5:**
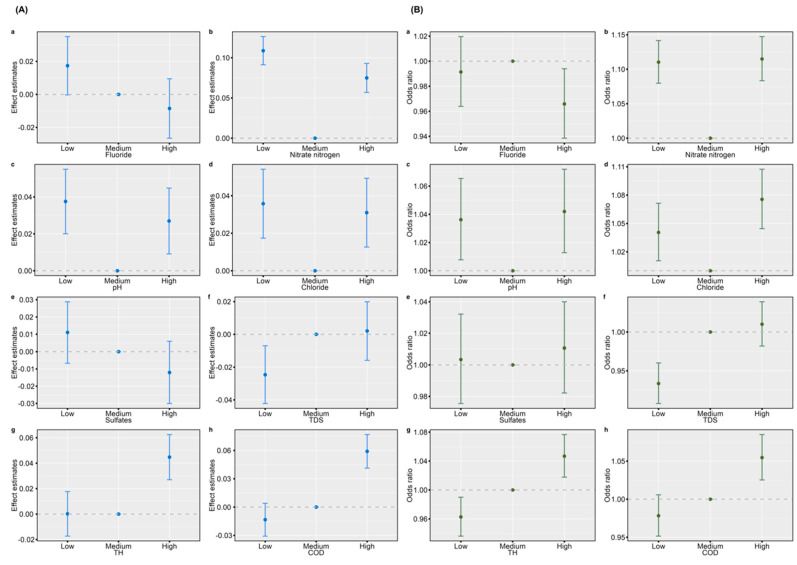
Associations of regular drinking water indicators with BMI Z-score and OB of all participants. Note: (**A**), BMI Z-score; (**B**), OB; (**a**) fluoride; (**b**) nitrate nitrogen; (**c**) pH; (**d**) chloride; (**e**) sulfates; (**f**) TDS; (**g**) TH; (**h**) COD; OB, overweight and obesity; TDS, total dissolved solids; TH, total hardness; COD, chemical oxygen demand. Estimates were adjusted for sex, age, city-rural water, vegetables, fruits, sugared beverages, fried food, physical activity time, outdoor time, sleeping time, smoking, and drinking.

As shown in Figure 4, the RCS curves demonstrated that fluoride, nitrate nitrogen, and pH had nonlinear (“U-shape”) dose-response associations with BMI Z-score (fluoride: P for nonlinear < 0.001, nitrate nitrogen: P for nonlinear < 0.001, pH: P for nonlinear < 0.001). Specifically, both high and low fluoride, nitrate nitrogen, and pH levels were associated with increased BMI Z-scores. Our research also observed that chloride, sulfates, and TDS were associated with significant decreases in BMI Z-scores in children and adolescents. In addition, the exposure-response curves revealed that certain water indicator factors (nitrate nitrogen and pH) exhibit a “U-shape” dose-response relationship with OB, as indicated by their nonlinear patterns. Both low and high nitrate nitrogen and pH levels were associated with increased odds of OB. Moreover, our study highlighted that TH and COD were positively associated with OB. Additionally, we observed that certain water indicators, such as fluoride, chloride, sulfates, and TDS, did not exhibit a significant association with OB (*p* > 0.05). Figure 5 illustrates similar associations between drinking water indicators and BMI Z-score as well as OB for all participants when using the second quartile (T2) as a baseline reference.

Stratified analyses according to participants’ sex and living regions were shown in Figures S2–S9, and similar associations across these diverse demographic categories were witnessed. Significantly, we observed that both increases and decreases in pH levels were associated with elevated BMI Z-scores among girls, while only increases in pH levels were linked to elevated BMI Z-scores among boys. As for urban-rural disparities, our findings revealed that elevated TH levels were associated with increased odds of OB among children and adolescents in urban regions but not in rural regions.

### 3.4. Modifying Effect of Lifestyle Behavior on the Association Between Drinking Water Micronutrients and BMI

**Figure 6:**
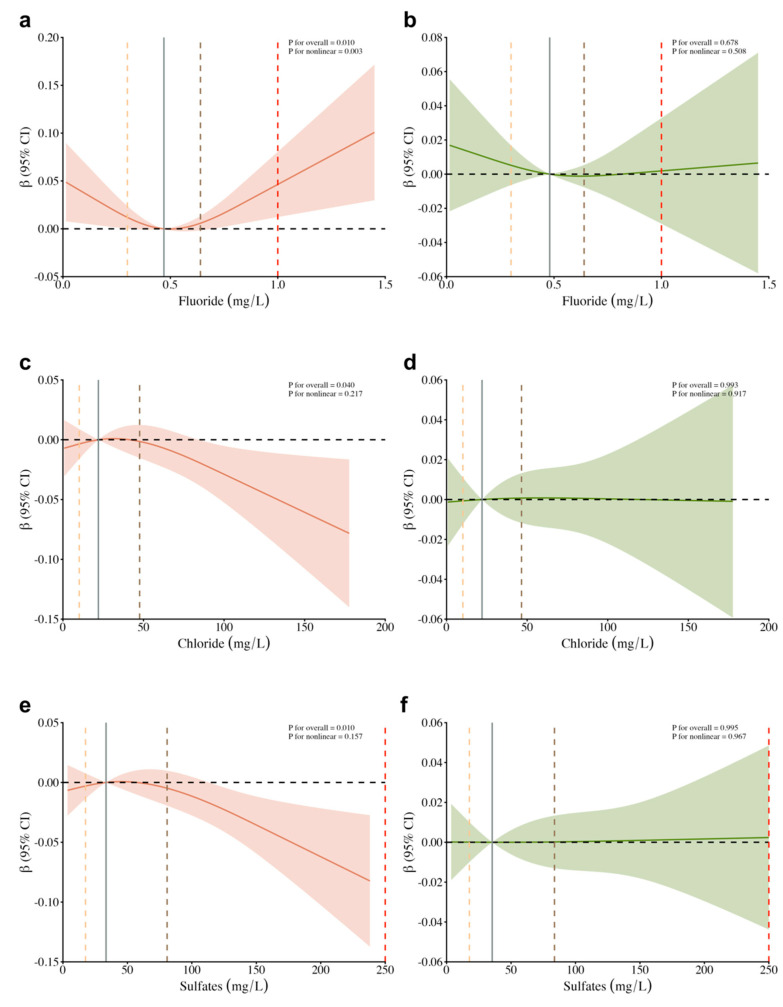
The moderating effect of lifestyle behavior on the associations of fluoride, chloride, and sulfates with BMI Z-score among all participants. Note: (**a**) fluoride-unhealthy lifestyles; (**b**) fluoride-healthy lifestyles; (**c**) chloride-unhealthy lifestyles; (**d**) chloride-healthy lifestyles; (**e**) sulfates-unhealthy lifestyles; (**f**) sulfates-healthy lifestyles; Solid lines were predicted curves; shadow parts were 95% confidence intervals. The orange, black, and grey lines indicate the 25th, median, and 75th concentration levels. The red broken lines indicate the water quality standard limit according to the administrative standard.

Figure 6 shows the effect modification by lifestyle behavior on the associations of fluoride, chloride, and sulfates with BMI Z-score among all participants. Figures S10 and S11 show the association between regular drinking water indicators and BMI by lifestyle behavior. The RCS curve indicated that fluoride, chloride, and sulfate levels significantly influence BMI Z-score among children and adolescents with unhealthy lifestyle behavior; however, this association is not observed within the group adhering to healthy lifestyle behavior. Meanwhile, no modifying effects of healthy lifestyle behavior on associations between drinking water indicators and OB were found.

## 4. Discussion

Using large-scale data from the population surveys, fluoride, nitrate nitrogen, and pH had nonlinear dose-response associations with BMI Z-score, and chloride, sulfates, and TDS were associated with significant decreases in BMI Z-scores. In particular, the current study only observed the association between fluoride and chloride with BMI Z-scores but not with OB. In girls, BMI Z-scores were associated with both high and low pH levels, whereas in boys, only the elevated pH levels showed a significant association. Additionally, only urban participants exhibited an association between increased TH levels and a heightened risk of OB. Furthermore, adopting healthy lifestyles mitigates the influence of fluoride, chloride, and sulfate levels on the BMI Z-scores of individuals. These findings provide a distinctive insight into the potential health advantages linked to specific water indicators in children and adolescents.

Our findings support a nonlinear association between fluoride, nitrate nitrogen, and pH levels with BMI. Increasing evidence suggests that high nitrate concentrations in drinking water may be linked to various health issues [27,28]. There is some suggestive evidence that children with elevated nitrate levels may face an increased risk of respiratory tract infections and goiter [29]. Similarly, pH levels exhibit a nonlinear association with BMI, which may reflect the body’s capacity to maintain homeostasis within certain boundaries of pH variation, beyond which adverse effects on BMI become manifest. Opting for high-pH drinking water may lower the likelihood of experiencing diarrhea compared to consuming other unimproved water sources [30]. These are inconsistent with the results of our study, which found that both high and low pH levels could increase OB risk in girls. The underlying mechanisms remain unclear, while high or low pH may alter appetite, gastrointestinal function, or metabolism, with complex effects on the risk of OB [31,32]. Meanwhile, our study found that both increases and decreases in pH levels were associated with elevated BMI Z-scores among girls, while only increases in pH levels were linked to elevated BMI Z-scores among boys. The differential associations between pH levels and BMI Z-scores in boys and girls may be attributed to several factors. First, hormonal differences between genders may influence metabolic processes and fat distribution [33,34], potentially altering how pH levels affect body composition. For instance, estrogen in females is known to promote fat storage [33], which might interact with changes in pH to influence BMI Z-scores differently than in boys. Meanwhile, this research suggests that the gut microbiome composition can differ between genders, influencing metabolic health and BMI Z-scores [35]. Changes in pH levels might affect the microbiome differently in boys and girls, potentially impacting nutrient absorption and energy metabolism.

Additionally, our research indicated that elevated TH in water is associated with increased BMI, particularly in urban populations. Studies also have found that areas with higher TH levels have a higher incidence of cardiovascular disease [36]. The association between TH in water and increased BMI may be interpreted through the lens of mineral metabolism and its potential disruption [37]. Existing research suggests that TH, such as calcium (Ca) and magnesium (Mg) content in drinking water, may play a preventive role in the initial stages of atherosclerosis in children and adolescents [38]. Furthermore, the acceleration of urbanization and climate change has had a growing association with the health of urban dual water cycles [39,40,41]. Meanwhile, we found an inverse association between chloride levels and BMI Z-scores. One possible explanation for this inverse relationship could be derived from the diuretic effect associated with increased chloride intake, leading to a reduction in extracellular fluid volume and, thus, potentially a lower body weight.

Our study revealed an inverse association between chloride levels and BMI Z-scores. This finding suggests that as chloride levels increase, there is a tendency for body weight to decrease. The finding is consistent with the current findings [42,43,44]. The underlying mechanism may involve perchlorate acting as an iodide symporter inhibitor, which can disrupt iodide uptake into the thyroid and consequently impair thyroid function [45]. However, it is important to note that studies on perchlorate-related thyroid disorders have yielded conflicting results. For instance, several studies reported that exposure to low levels of perchlorate does not significantly affect thyroid function in pregnant women, even in iodine-rich regions [46,47]. Meanwhile, other studies reported development issues in infants from areas with perchlorate contamination in drinking water [48,49]. Another possible explanation for this inverse relationship could be derived from the diuretic effect associated with increased chloride intake, leading to a reduction in extracellular fluid volume and, thus, potentially a lower body weight. In addition, our study found that when the drinking water indicators are lower than the water standard quality limits, they can still be associated with the BMI of children and adolescents. The observed association could indicate that even minimal levels of certain contaminants in drinking water may have metabolic effects on growing bodies [50]. Children and adolescents are particularly vulnerable to environmental factors due to their developing physiology and the higher consumption of water compared to adults [50,51]. Therefore, this emphasizes the importance of re-evaluating drinking water indicator standards and developing comprehensive strategies to ensure the well-being of younger populations.

Current research underscores the significant benefits of healthy lifestyle behavior, demonstrating its capacity to diminish the effect of fluoride, chloride, and sulfate on the BMI Z-scores of children and adolescents. The modifying effects of healthy lifestyle behavior may be attributed to several mechanisms. Firstly, regular physical activity, a fundamental component of a healthy lifestyle, has enhanced metabolic health and increased energy expenditure, potentially counteracting the weight-promoting effects of environmental toxins [52,53]. Moreover, the antioxidant and anti-inflammatory properties of a nutrient-dense diet may help mitigate oxidative stress and inflammation that these chemicals can induce, thereby preventing obesity associated with such physiological disturbances [54,55]. Our study underscores the potential for healthy lifestyle behavior to act as modifiable factors, diminishing the adverse effects of fluoride, chloride, and sulfate on BMI. Consequently, these findings contribute valuable insights to the discourse on pediatric environmental health and disease prevention.

Our study has several strengths over previous studies. First and most importantly, we employed a systematic and comprehensive assessment approach, coupled with highly accurate monitoring of common diseases and drinking water indicators. The validity of our surveillance system is continuously monitored to ensure its effectiveness. Secondly, the present study provides the scientific rationale and epidemic-logic evidence among the large population to directly infer the association between perchlorate in drinking water and children and adolescents’ growth. Thirdly, we performed a sensitivity analysis to verify the robustness of our results and understand that drinking water indicators may influence BMI in various demographic subgroups.

However, it is important to acknowledge certain limitations of our study. Notably, the study population was limited to students within a large regional surveillance system, which does not include children and adolescents outside the school system. In addition, our study did not investigate the amount or the sources of their water consumption, which may limit the precision of our findings on the association between drinking water indicators and health outcomes. For instance, fluoride and chloride intake may be influenced by dietary sources such as soft drinks [56]. Further studies should consider a more comprehensive assessment of the exposure from dietary sources to better understand their effects on BMI. Meanwhile, due to the national cross-sectional design of our study, we are unable to establish a causal relationship between drinking water indicators and BMI. Furthermore, factors such as genetics, medication use, and other environmental variables can also affect the height and weight of children and adolescents.

## 5. Conclusions

In conclusion, this large surveillance study provides new insights into the associations between various drinking water parameters and BMI. Meanwhile, healthy lifestyle behavior could mitigate the negative effects of fluoride, chloride, and sulfate on BMI. This study emphasizes the need to further explore water indicators’ impacts on growth, particularly in urban environments, and highlights the importance of adopting healthy lifestyles to mitigate adverse effects. These insights could guide the refinement of future drinking water standards and offer actionable lifestyle interventions to enhance the health of children and adolescents.

## Figures and Tables

**Table 1:** Characteristics of the participants included in this study.

Variables		Total	Non-OB	OB	*p*-Value
Sex	Boys	87,676 (50.71)	45,520 (44.53)	42,156 (59.65)	<0.001
	Girls	85,204 (49.29)	56,692 (55.47)	28,512 (40.35)	
Age	Age (years)	12.09 ± 2.98	12.33 ± 3.08	11.74 ± 2.81	<0.001
	7~12 years	103,617 (59.94)	56,773 (55.54)	46,844 (66.29)	<0.001
	13~15 years	47,980 (27.75)	30,444 (29.79)	17,536 (24.81)	
	16~17 years	21,283 (12.31)	14,995 (14.67)	6288 (8.90)	
	Height	152.30 ± 15.89	151.80 ± 16.34	152.90 ± 15.19	<0.001
	Weight	49.30 ± 17.95	42.50 ± 13.21	59.10 ± 19.33	<0.001
	BMI (kg/m^2^)	20.63 ± 4.67	17.92 ± 2.45	24.56 ± 4.32	<0.001
	BMI Z-score	0.72 ± 1.37	−0.21 ± 0.84	2.06 ± 0.73	<0.001
Vegetables	Never	5349 (4.17)	3306 (4.31)	2043 (3.97)	<0.001
	<1 time	21,004 (16.38)	12,958 (16.88)	8046 (15.63)	
	1 time	48,278 (37.65)	28,874 (37.62)	19,404 (37.69)	
	≥2 times	53,611 (41.8)	31,615 (41.19)	21,996 (42.72)	
Fruits	Never	7376 (5.75)	4528 (5.90)	2848 (5.53)	<0.001
	<1 time	35,350 (27.57)	21,981 (28.64)	13,369 (25.96)	
	1 time	56,001 (43.67)	33,192 (43.25)	22,809 (44.30)	
	≥2 times	29,515 (23.02)	17,052 (22.22)	12,463 (24.21)	
Sugared beverages	Never	46,255 (36.07)	27,536 (35.88)	18,719 (36.36)	0.001
	<1 time	75,512 (58.88)	45,199 (58.89)	30,313 (58.87)	
	≥1 time	6475 (5.05)	4018 (5.23)	2457 (4.77)	
Fried food	Never	43,017 (33.54)	25,594 (33.35)	17,423 (33.84)	<0.001
	<1 time	80,262 (62.59)	48,025 (62.57)	32,237 (62.61)	
	≥1 time	4963 (3.87)	3134 (4.08)	1829 (3.55)	
Physical activity time	0~2 days	55,161 (43.01)	33,072 (43.09)	22,089 (42.90)	<0.001
	3~5 days	46,012 (35.88)	27,173 (35.40)	18,839 (36.59)	
	6~7 days	27,069 (21.11)	16,508 (21.51)	10,561 (20.51)	
Outdoor time	<2 h	76,447 (64.68)	46,016 (65.15)	30,431 (64.00)	<0.001
	≥2 h	41,737 (35.32)	24,619 (34.85)	17,118 (36.00)	
Sleeping time	≤7 h	36,352 (28.35)	23,742 (30.93)	12,610 (24.49)	<0.001
	8~9 h	60,858 (47.46)	35,730 (46.55)	25,128 (48.80)	
	≥9 h	31,032 (24.20)	17,281 (22.52)	13,751 (26.71)	
Smoking	Yes	5916 (4.61)	3793 (4.94)	2123 (4.12)	<0.001
	No	12,232 6(95.39)	72,960 (95.06)	49,366 (95.88)	
Alcohol drinking	Yes	15,371 (11.99)	9464 (12.33)	5907 (11.47)	<0.001
	No	112,871 (88.01)	67,289 (87.67)	45,582 (88.53)	

Note: Non-OB, non-overweight and obesity; OB, overweight and obesity; BMI, body mass index. The subgroup differences between non-OB and OB groups were examined using a *t*-test (continuous variables) or Chi-squared test (categorical variables).

## Data Availability

The data presented in this study are available on request from the corresponding author due to the legal protection by the Inner Mongolia Center for Disease Control and Prevention.

## Supplementary Materials

The following supporting information can be downloaded at: https://www.mdpi.com/article/10.3390/nu16223931/s1, Method S1. Detailed information on the water sampling and examinations. Table S1. Characteristics of regular indices for drinking water under different nutritional status. Table S2. Characteristics of regular indices for drinking water between urban and rural areas. Figure S1. The comparison of regular drinking water indicators between non-OB and OB groups. Figure S2. Exposure-response curves for the associations of regular drinking water indicators with BMI Z-score and OB of boys. Figure S3. Exposure-response curves for the associations of regular drinking water indicators with BMI Z-score and OB of girls. Figure S4. Exposure-response curves for the associations of regular drinking water indicators with BMI Z-score and OB of urban participants. Figure S5. Exposure-response curves for the associations of regular drinking water indicators with BMI Z-score and OB of rural participants. Figure S6. Associations of regular drinking water indicators with BMI Z-score and OB of boys. Figure S7. Associations of regular drinking water indicators with BMI Z-score and OB of girls. Figure S8. Associations of regular drinking water indicators with BMI Z-score and OB of urban participants. Figure S9. Associations of regular drinking water indicators with BMI Z-score and OB of rural participants. Figure S10. Exposure-response curves for the associations of regular drinking water indicators with BMI Z-score of all participants, stratified by lifestyle behavior. Figure S11. Exposure-response curves for the associations of regular drinking water indicators with OB of all participants stratified by lifestyle behavior.
