# Supplementary material for: The Modifying Effects of Lifestyle Behaviors on the Association Between Drinking Water Micronutrients and BMI Status Among Children and Adolescents Aged 7~17: A Population-Based Regional Surveillance in 2022
Source: Nutrients. 2024 Nov 18;16(22):3931. doi: 10.3390/nu16223931 (PMC11597896; doi:10.3390/nu16223931)
Supplement: Supplementary file 1 [file nutrients-16-03931-s001.zip › nutrients-3291310-supplementary.pdf]

List of Supplementary Materials

Table of Contents:

Method S1. Detailed information on the water sampling and examinations.

Table S1. Characteristics of regular indices for drinking water under different nutritional status

Table S2. Characteristics of regular indices for drinking water between urban and rural areas

Figure S1. The comparison of regular drinking water indicators between Non-OB and OB groups

Figure S2. Exposure-response curves for the associations of regular drinking water indicators with BMI Z-score and OB of boys

Figure S3. Exposure-response curves for the associations of regular drinking water indicators with BMI Z-score and OB of girls

Figure S4. Exposure-response curves for the associations of regular drinking water indicators with BMI Z-score and OB of urban participants

Figure S5. Exposure-response curves for the associations of regular drinking water indicators with BMI Z-score and OB of rural participants

Figure S6. Associations of regular drinking water indicators with BMI Z-score and OB of boys

Figure S7. Associations of regular drinking water indicators with BMI Z-score and OB of girls

Figure S8. Associations of regular drinking water indicators with BMI Z-score and OB of urban participants

Figure S9. Associations of regular drinking water indicators with BMI Z-score and OB of rural participants

Figure S10. Exposure-response curves for the associations of regular drinking water indicators with BMI Z-score of the all participants, stratified by lifestyle behaviors

Figure S11. Exposure-response curves for the associations of regular drinking water indicators with OB of the all participants, stratified by lifestyle behaviors

Method S1. Detailed information on the water sampling and examinations.

Sampling methods:

Sampling Containers: For sample collection, narrow-mouth containers should be used, and the materials of the lids and stoppers must match those of the containers. For the determination of inorganic substances, metals, and radioactive elements, sampling containers made of organic materials, such as polyethylene plastic, were utilized. For samples intended for the analysis of organic compounds and microbiological indicators, glass containers were used.

Cleaning of Sampling Containers: The washing procedure for containers used for measuring general physicochemical indicators involves rinsing the containers with water and detergent to remove dust and grease, followed by thorough rinsing with tap water. Subsequently, the containers should be soaked in a 10% nitric acid (or hydrochloric acid) solution for 8 hours. After soaking, they should be drained and rinsed three times with tap water, followed by extensive rinsing with distilled water.

Collection of Terminal Water Samples: Attention should be paid to the timing of sample collection. At night, sediments that may settle in the pipes can be released. Therefore, the tap should be opened and water should be allowed to flow for several minutes to flush out any deposits. Before sampling, the sampler, container, and stopper should be rinsed 2–3 times with the water sample. For measuring dissolved oxygen and biochemical oxygen demand, the container should be filled with no headspace left, and a water seal should be used.

Detection methods:

(1) Fluoride, nitrate, chloride, sulfate: ion chromatography

Water samples containing the anions of interest are introduced into an ion exchange column system, which consists of a guard column and a separation column. The separation column differentiates the anions based on their varying affinities, allowing for their separation. The eluted anions then pass through either a cation exchange column or a suppressor system, converting them into strong acids with high conductivity, while the eluent is transformed into a weakly conductive carbonate. The conductivity detector measures the conductivity of each anion component, allowing for qualitative and quantitative analysis based on relative retention times and peak heights or areas.

Instrument Preparation: Turn on the ion chromatography system. Adjust the flow rates of the eluent and regenerant according to the instrument manual to achieve equilibrium and a stable baseline.

Calibration: Inject a mixed anion standard solution along with two serially diluted standards of three different concentrations into the sample introduction system. Plot the peak heights or areas to create a calibration curve.

Sample Analysis:

Pre-treatment: Filter the water sample through a 0.2-micrometer membrane to remove turbidity. For samples with high hardness, it may be necessary to pass the sample through a cation exchange resin column before filtering. Samples containing organic matter should be filtered through a carbon column to remove impurities.

Inject the pre-treated water sample into the chromatography system and record the peak heights or areas.

Calculation: The mass concentration of various anions (in mg/L) can be determined directly from the calibration curve.

(1) pH levels: glass electrode method

Prior to use: the glass electrode should be immersed in pure water for more than 24 hours.
Instrument Calibration: After turning on the instrument, allow it to warm up for 30 minutes before following the operational instructions provided in the user manual.
pH Measurement: Select a standard buffer solution that is close in pH to the sample being tested. Repeat the calibration process one to two times. If the sample pH is less than seven, use a potassium hydrogen phthalate standard buffer for calibration, followed by a sodium tetraborate or mixed phosphate buffer for subsequent calibration. Conversely, if the sample pH is greater than seven, use a sodium tetraborate standard buffer initially, followed by potassium hydrogen phthalate or mixed phosphate buffer for recalibration. Note: If the calibration values from the three buffer solutions do not show linearity, the quality of the glass electrode should be checked.
Rinse both electrodes several times with pure water using a wash bottle, followed by rinsing with the sample water six to eight times. Then, immerse the electrode in the sample and read the pH value directly from the instrument after one minute.

(3) total hardness (TH): disodium ethylene-diamine-tetra-acetate (EDTA) titration method
Take a 50-milliliter sample of water (if the hardness is excessively high, dilute the sample with pure water to achieve a total volume of 50 milliliters; if it is too low, use a 100-milliliter sample) and place it in a 150-milliliter conical flask.
Add one to two milliliters of buffer solution and five drops of chrome black T indicator. Immediately titrate with a standard NaEDTA solution until the solution changes from purplish-red to pure blue, concurrently conducting a blank test to record the volume used.
If the sample contains metallic interference ions that delay the endpoint or darken the color, take a new sample and add 0.5 milliliters of hydroxylamine hydrochloride and one milliliter of sodium sulfide solution or 0.5 milliliters of potassium chromate solution, then proceed with titration.
If the sample contains significant amounts of calcium and magnesium bicarbonates, acidify the sample beforehand and heat to remove carbon dioxide to prevent the formation of carbonate precipitates during titration.
If the sample contains suspended or colloidal organic matter that may affect endpoint observation, evaporate the sample to dryness and ash at 550 degrees Celsius. Dissolve the residue in pure water before proceeding with titration.

(4) total dissolved solids (TDS): 105°C drying and weighing method
Preparation of Evaporating Dish: Clean the evaporating dish and place it in an oven set at a temperature of 105 degrees Celsius, allowing it to dry for approximately half an hour. Afterward, cool the dish in a desiccator for about half an hour.
Weight Measurement: Using an analytical balance, weigh the dish, then reheat and reweigh it until a constant mass is achieved (the difference between two measures should not exceed a minimal threshold).
Sample Preparation: Filter the supernatant of the water sample using a filter. Using a pipette, transfer 100 milliliters of the filtered water sample into the evaporating dish. If the dissolved solids content is low, increase the volume of the sample.
Evaporation: Place the evaporating dish in a water bath to evaporate the liquid (ensuring the water level does not touch the bottom of the dish). After one hour in the 105-degree oven, remove the dish and cool it again in a desiccator for 30 minutes before weighing.

Final Weight Measurement: Return the weighed evaporating dish to the oven for another 30 minutes, cool it in the desiccator, and weigh it until a constant mass is obtained.

(5) Chemical Oxygen Demand (COD): the acidic or alkaline potassium permanganate titration method

Preparation of Erlenmeyer Flask: Add a milliliter of sulfuric acid solution and a small amount of potassium permanganate standard solution to a 250-milliliter Erlenmeyer flask. Boil for a few minutes, then titrate with sodium oxalate standard solution until a faint red color appears, and discard the solution.

Sample Analysis: Transfer 100 milliliters of the thoroughly mixed water sample (if the organic matter content is high, dilute to 100 milliliters using pure water) into the previously treated Erlenmeyer flask. Add five milliliters of sulfuric acid solution. Use a burette to add ten milliliters of potassium permanganate standard solution.

Heating the Sample: Place the Erlenmeyer flask in a boiling water bath for exactly 30 minutes. If the red color significantly fades during heating, the sample must be diluted and reanalyzed.

Titration: Remove the flask from the water bath and, while still warm, add ten milliliters of sodium oxalate standard solution, shaking thoroughly to decolorize the solution. On a white background, use the burette to add potassium permanganate standard solution until a faint red color reappears; record the volume used.

Table S1. Characteristics of regular indices for drinking water under different nutritional status

Indices for drinking water	Total		Non-OB	OB	*P* value
	Limit	M (*P*25, *P*75)	M (*P*25, *P*75)	M (*P*25, *P*75)	
Fluoride, mg/L	1	0.48(0.30, 0.67)	0.48(0.30, 0.67)	0.50(0.30, 0.67)	0.812
Nitrate nitrogen, mg/L	10	3.00(0.80, 6.96)	2.96(0.93, 6.96)	3.00(0.80, 7.00)	<0.001
pH	6.5～8.5	7.50(7.24, 7.81)	7.50(7.24, 7.80)	7.50(7.23, 7.82)	0.392
Chloride, mg/L	250	25.50(10.40, 63.00)	25.50(10.75, 63.00)	25.30(10.20, 63.00)	0.002
Sulfates, mg/L	250	35.42(17.80, 93.27)	35.42(17.80, 90.10)	35.81(17.80, 93.27)	<0.001
Total dissolved solids, mg/L	1000	326.00(252.00, 531.00)	326.00(249.00, 531.00)	328.00(252.00, 531.40)	<0.001
Total hardness, mg/L	450	214.50(144.10, 286.97)	212.00(142.03, 286.20)	220.00(145.73, 288.00)	<0.001
COD, mg/L	3	0.96(0.56, 1.44)	0.96(0.56, 1.42)	0.98(0.56, 1.45)	<0.001

Note: Limit denotes the water quality standard limits according to the GB/T 5750-2006 and GB/T 5749-2006.

Table S2. Characteristics of regular indices for drinking water in urban and rural areas

Indices for drinking water	Urban (N=128,343)	Rural(N=44,537)	*P* value
	M (*P*25, *P*75)	M (*P*25, *P*75)	
Fluoride, mg/L	0.49(0.32, 0.64)	0.48(0.25, 0.70)	0.572
Nitrate nitrogen, mg/L	2.73(0.80, 6.10)	4.14(0.90, 7.40)	<0.001
pH	7.52(7.26, 7.84)	7.42(7.18, 7.70)	<0.001
Chloride, mg/L	29.00(10.89, 73.26)	22.20(9.12, 46.50)	<0.001
Sulfates, mg/L	38.50(18.56, 107.64)	33.43(11.30, 61.85)	<0.001
Total dissolved solids, mg/L	326.00(252, 512)	347.00(206.00, 539.00)	0.095
Total hardness, mg/L	218.20(144.10, 286.20)	196.18(141.00, 296.00)	0.033
COD, mg/L	0.96(0.55, 1.39)	1.02(0.66, 1.50)	<0.001

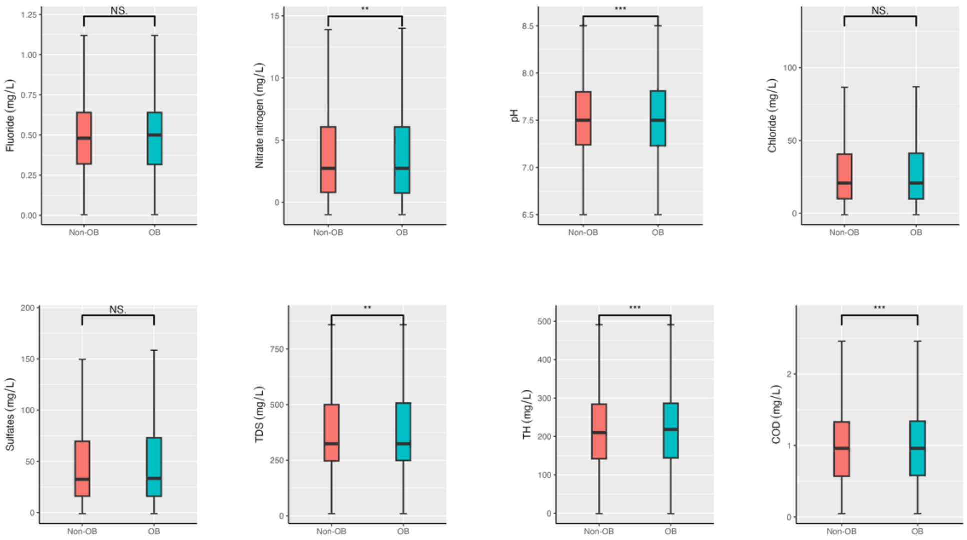

Figure S1. The comparison of regular drinking water indicators between Non-OB and OB groups

Note: Non-OB, non-overweight and obesity; OB, overweight and obesity; TDS, Total dissolved solids; TH, Total hardness; COD, Chemical oxygen demand.

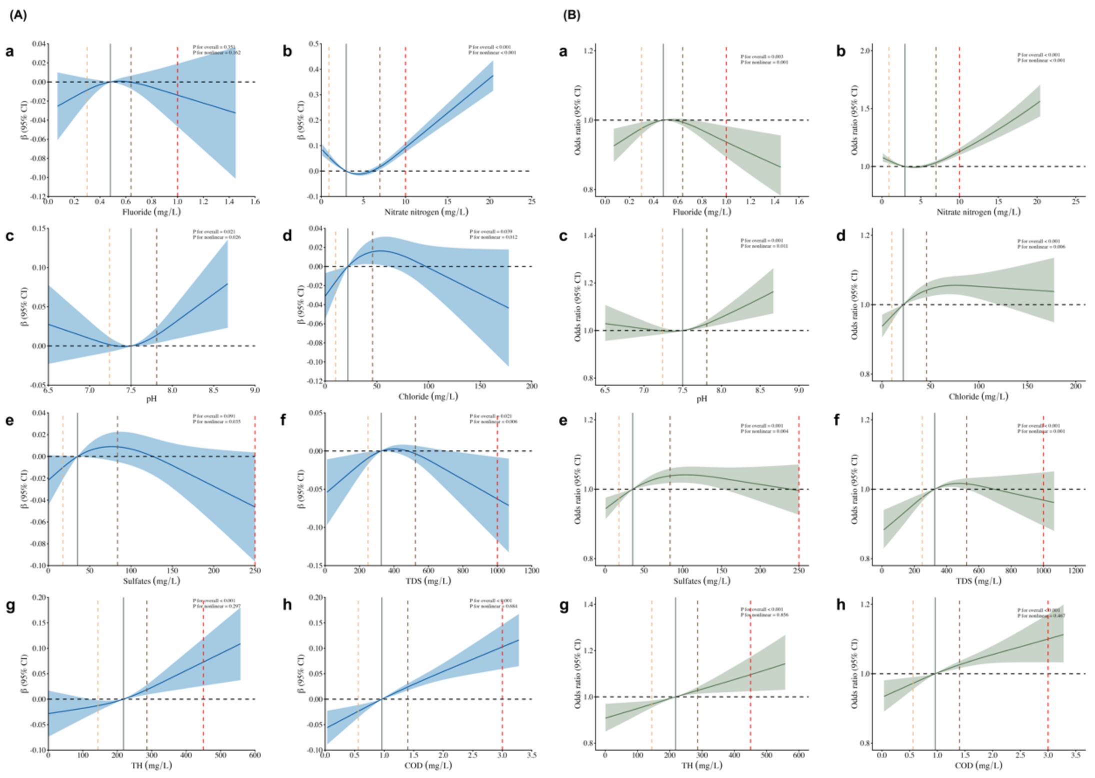

Figure S2. Exposure-response curves for the associations of regular drinking water indicators with BMI Z-score and OB of boys

Note: (A), BMI Z-score; (B), OB; OB, overweight and obesity; TDS, Total dissolved solids; TH, Total hardness; COD, Chemical oxygen demand. Estimates were adjusted for sex, age, and city-rural water, vegetables, fruits, sugared beverages, fried food, mid-high sports time, outdoor time, sleeping time, smoking, and drinking. Solid lines were predicted curves, shadow parts were 95% confidence intervals. The orange, black, and grey lines indicate the 25th, median, and 75th of the concentration levels. The red broken lines indicate the water quality standard limits according to the administrative standard.

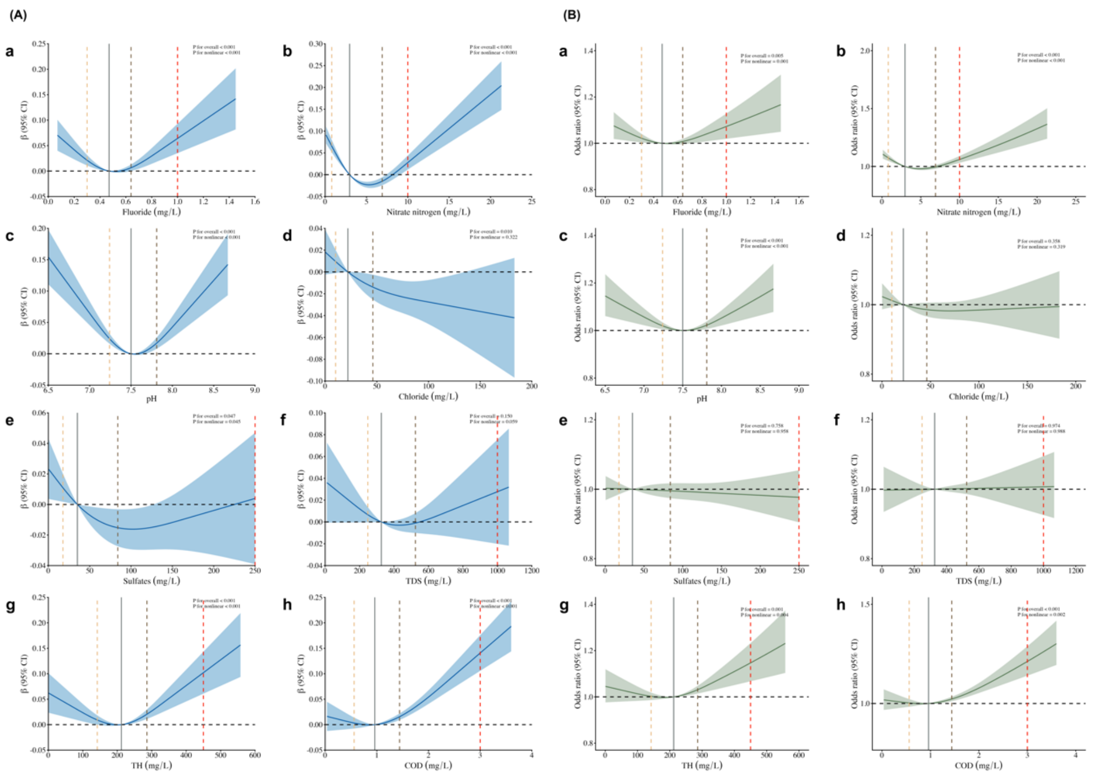

Figure S3. Exposure-response curves for the associations of regular drinking water indicators with BMI Z-score and OB of girls

Note: (A), BMI Z-score; (B), OB; OB, overweight and obesity; TDS, Total dissolved solids; TH, Total hardness; COD, Chemical oxygen demand. Estimates were adjusted for sex, age, and city-rural water, vegetables, fruits, sugared beverages, fried food, mid-high sports time, outdoor time, sleeping time, smoking, and drinking. Solid lines were predicted curves, shadow parts were 95% confidence intervals. The orange, black, and grey lines indicate the 25th, median, and 75th of the concentration levels. The red broken lines indicate the water quality standard limits according to the administrative standard.

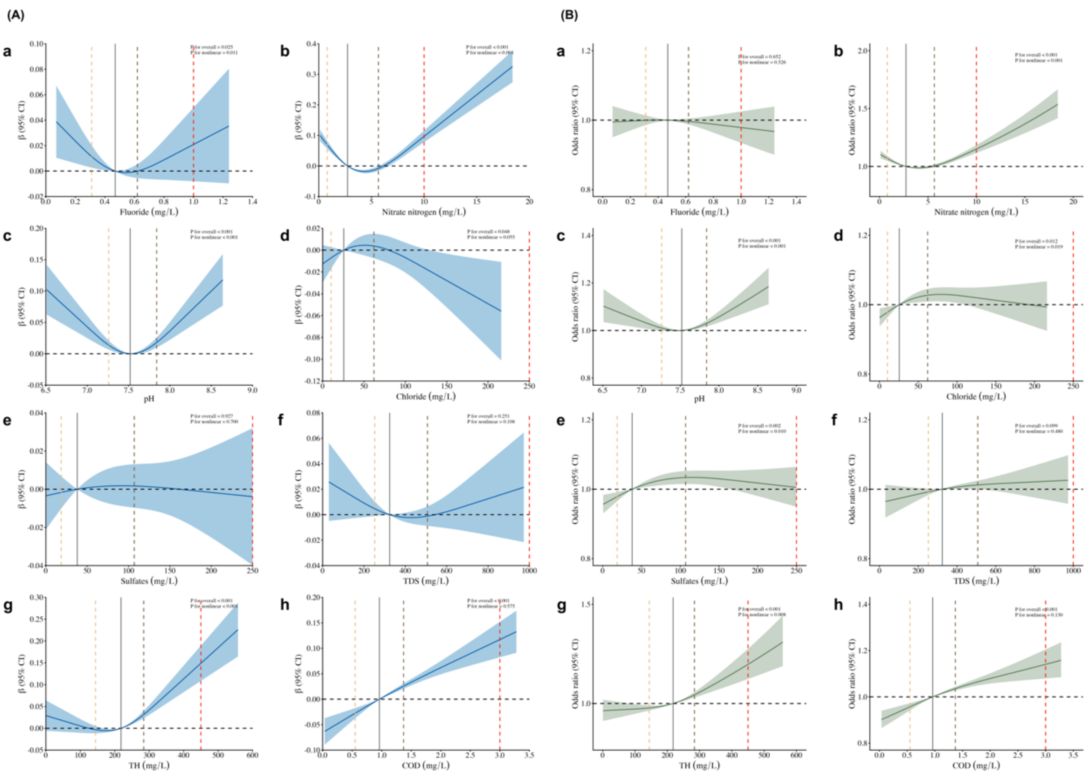

Figure S4. Exposure-response curves for the associations of regular drinking water indicators with BMI Z-score and OB of urban participants

Note: (A), BMI Z-score; (B), OB; OB, overweight and obesity; TDS, Total dissolved solids; TH, Total hardness; COD, Chemical oxygen demand. Estimates were adjusted for sex, age, and city-rural water, vegetables, fruits, sugared beverages, fried food, mid-high sports time, outdoor time, sleeping time, smoking, and drinking. Solid lines were predicted curves, shadow parts were 95% confidence intervals. The orange, black, and grey lines indicate the 25th, median, and 75th of the concentration levels. The red broken lines indicate the water quality standard limits according to the administrative standard.

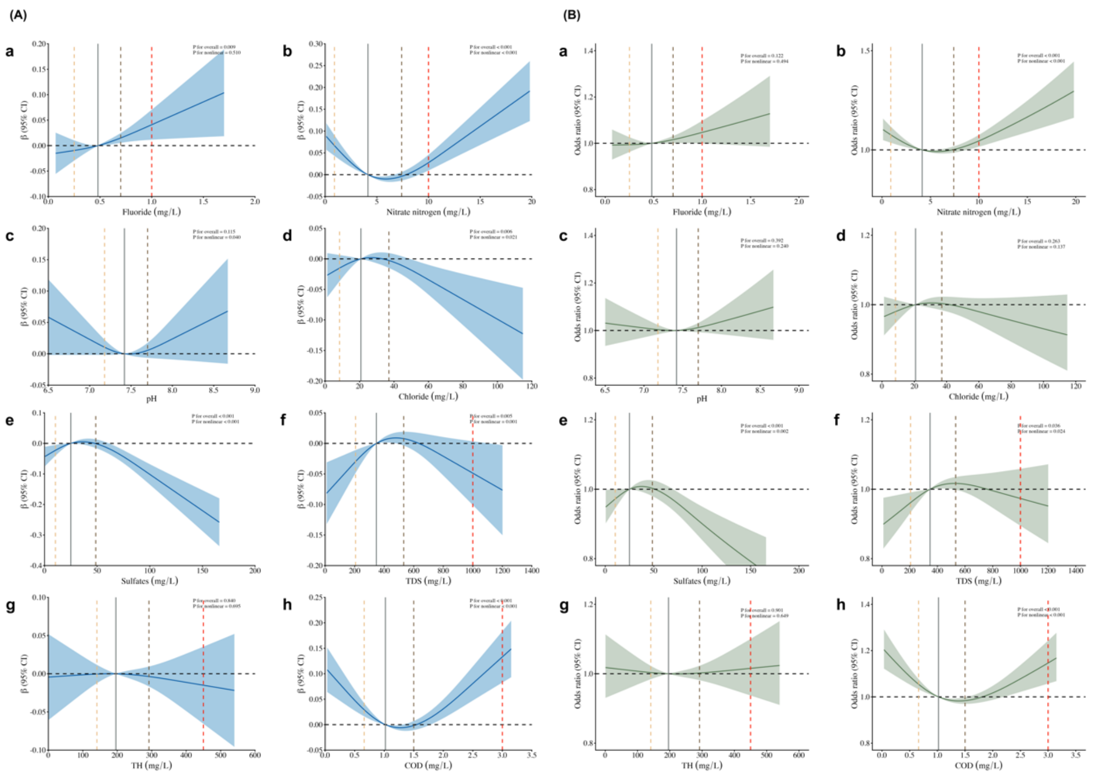

Figure S5. Exposure-response curves for the associations of regular drinking water indicators with BMI Z-score and OB of rural participants

Note: (A), BMI Z-score; (B), OB; OB, overweight and obesity; TDS, Total dissolved solids; TH, Total hardness; COD, Chemical oxygen demand. Estimates were adjusted for sex, age, and city-rural water, vegetables, fruits, sugared beverages, fried food, mid-high sports time, outdoor time, sleeping time, smoking, and drinking. Solid lines were predicted curves, shadow parts were 95% confidence intervals. The orange, black, and grey lines indicate the 25th, median, and 75th of the concentration levels. The red broken lines indicate the water quality standard limits according to the administrative standard.

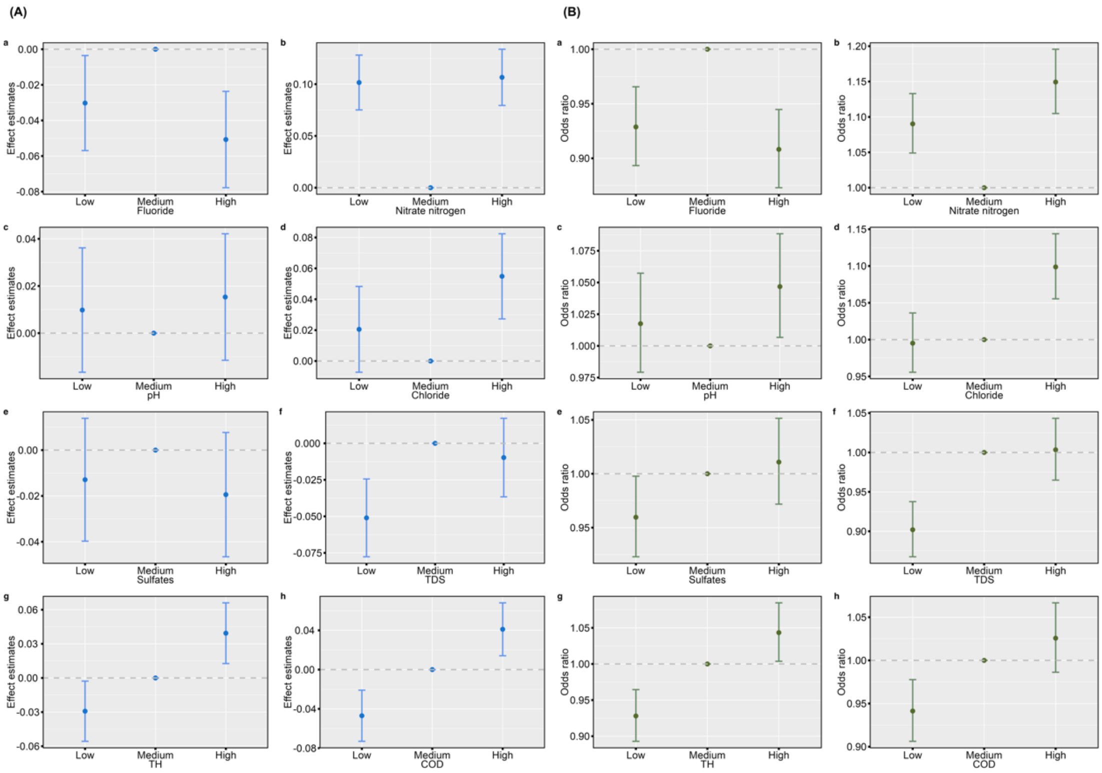

Figure S6. Associations of regular drinking water indicators with BMI Z-score and OB of boys

Note: (A), BMI Z-score; (B), OB; OB, overweight and obesity; TDS, Total dissolved solids; TH, Total hardness; COD, Chemical oxygen demand. Estimates were adjusted for sex, age, and city-rural water, vegetables, fruits, sugared beverages, fried food, mid-high sports time, outdoor time, sleeping time, smoking, and drinking.

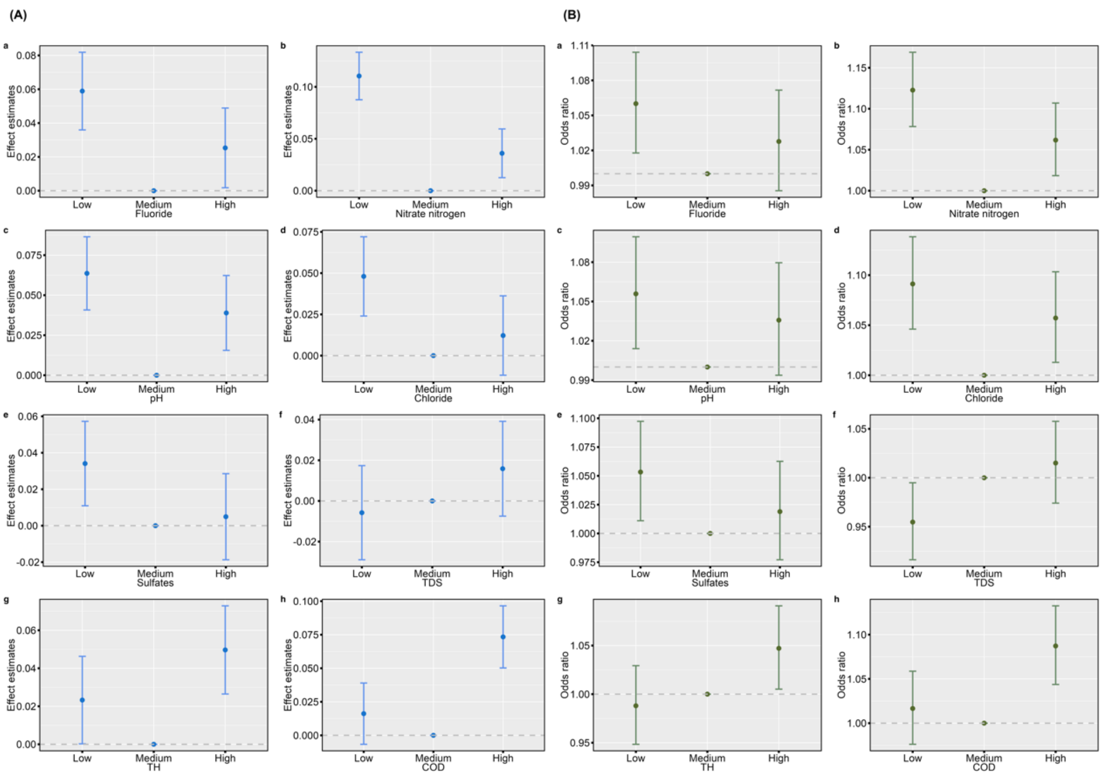

Figure S7. Associations of regular drinking water indicators with BMI Z-score and OB of girls

Note: (A), BMI Z-score; (B), OB; OB, overweight and obesity; TDS, Total dissolved solids; TH, Total hardness; COD, Chemical oxygen demand. Estimates were adjusted for sex, age, and city-rural water, vegetables, fruits, sugared beverages, fried food, mid-high sports time, outdoor time, sleeping time, smoking, and drinking.

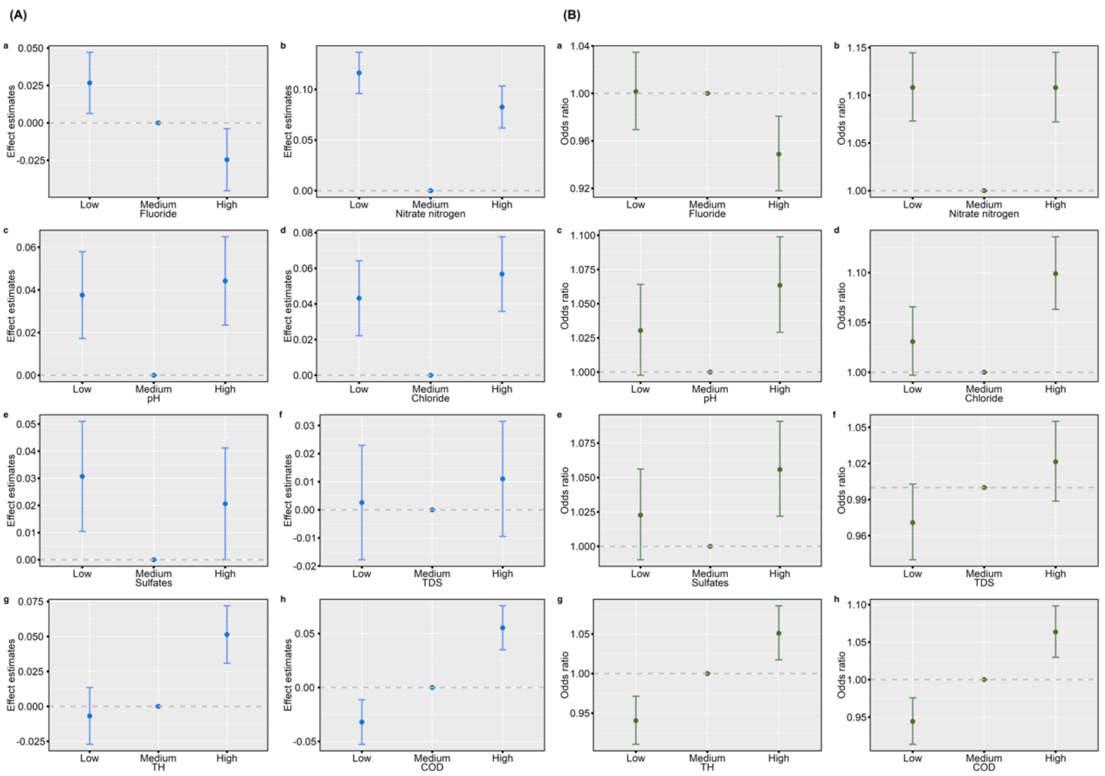

Figure S8. Associations of regular drinking water indicators with BMI Z-score and OB of urban participants

Note: (A), BMI Z-score; (B), OB; OB, overweight and obesity; TDS, Total dissolved solids; TH, Total hardness; COD, Chemical oxygen demand. Estimates were adjusted for sex, age, and city-rural water, vegetables, fruits, sugared beverages, fried food, mid-high sports time, outdoor time, sleeping time, smoking, and drinking.

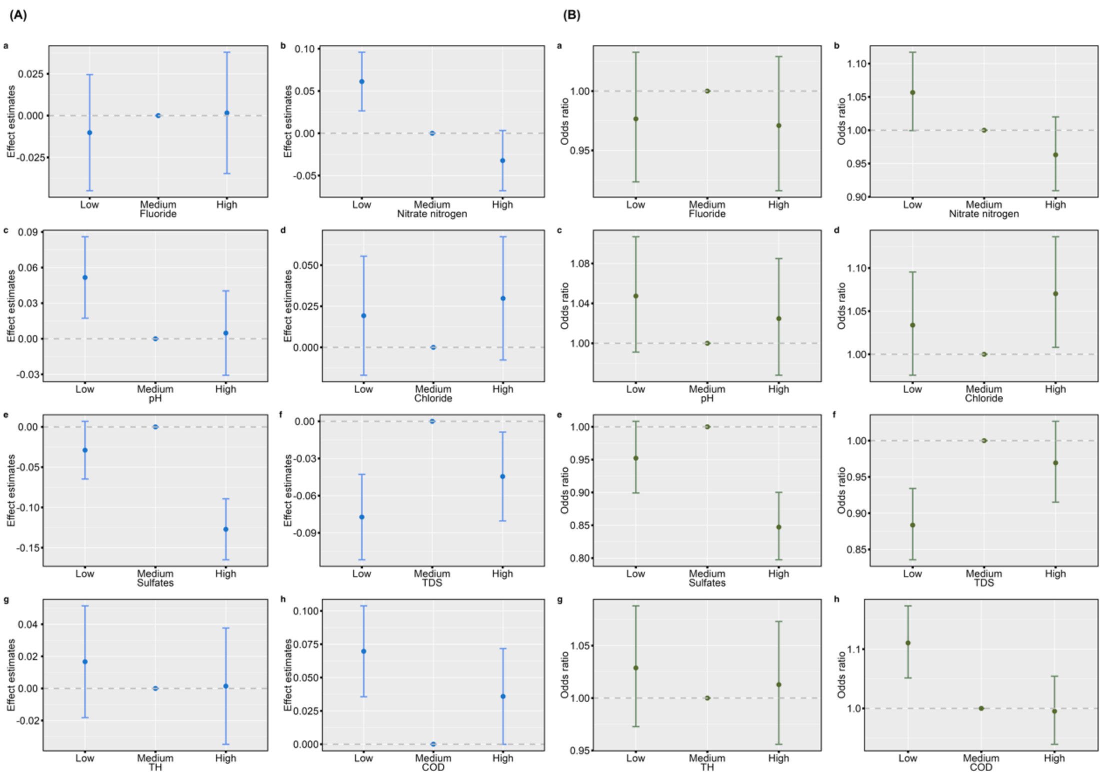

Figure S9. Associations of regular drinking water indicators with BMI Z-score and OB of rural participants

Note: (A), BMI Z-score; (B), OB; OB, overweight and obesity; TDS, Total dissolved solids; TH, Total hardness; COD, Chemical oxygen demand. Estimates were adjusted for sex, age, and city-rural water, vegetables, fruits, sugared beverages, fried food, mid-high sports time, outdoor time, sleeping time, smoking, and drinking.

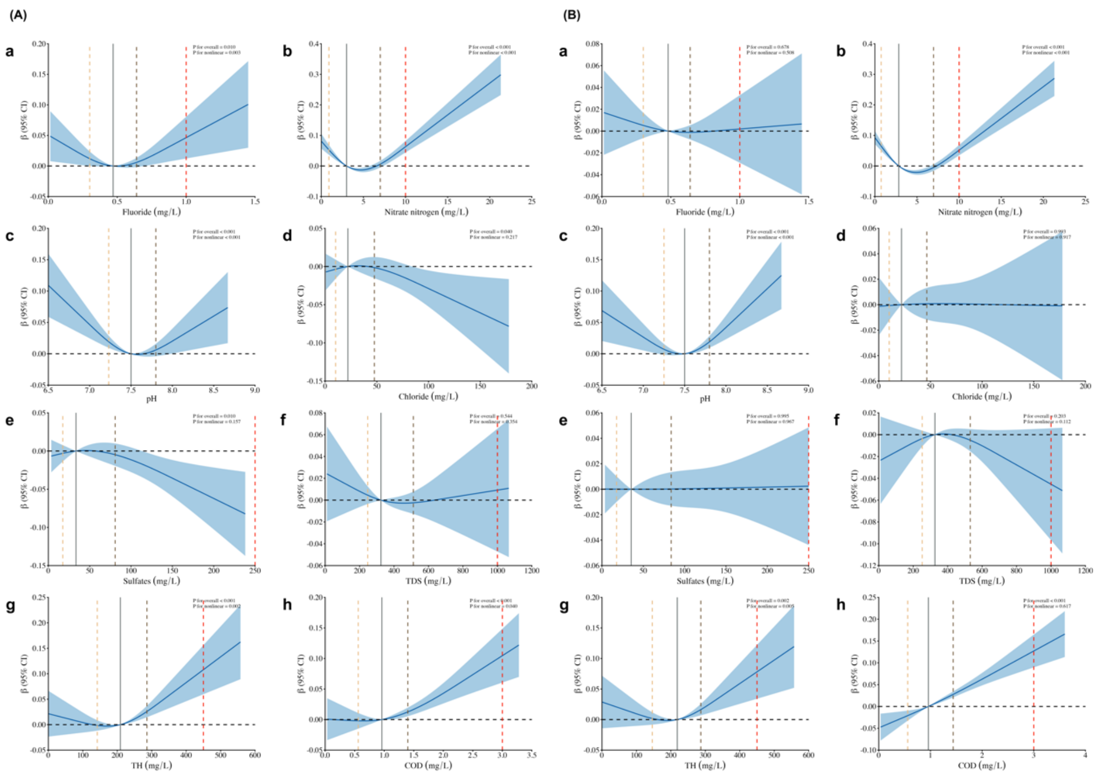

Figure S10. Exposure-response curves for the associations of regular drinking water indicators with BMI Z-score of the all participants, stratified by lifestyle behaviors

Note: (A), unhealthy lifestyle; (B), healthy lifestyle; Solid lines were predicted curves, shadow parts were 95% confidence intervals. The orange, black, and grey lines indicate the 25th, median, and 75th of the concentration levels. The red broken lines indicate the water quality standard limits according to the administrative standard.

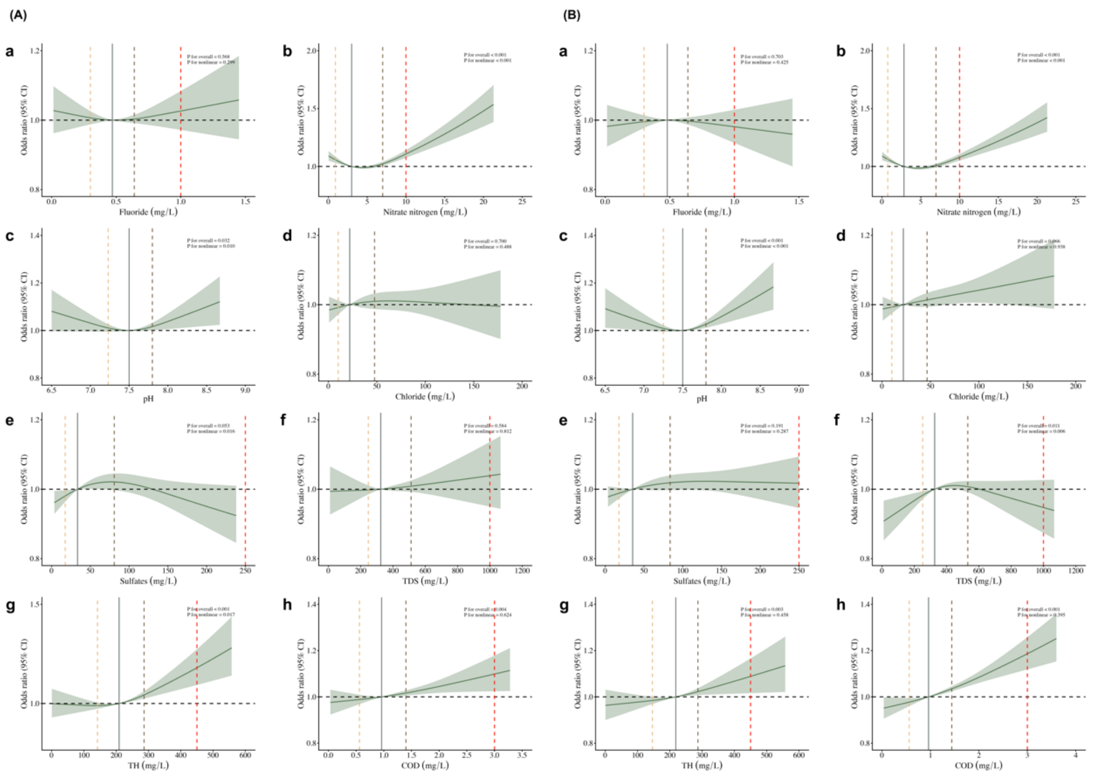

Figure S11. Exposure-response curves for the associations of regular drinking water indicators with OB of the all participants, stratified by lifestyle behaviors

Note: (A), unhealthy lifestyle; (B), healthy lifestyle; OB, overweight and obesity; Solid lines were predicted curves, shadow parts were 95% confidence intervals. The orange, black, and grey lines indicate the 25th, median, and 75th of the concentration levels. The red broken lines indicate the water quality standard limits according to the administrative standard.
